# Xylem morphology influences lemon susceptibility to mal secco disease

**DOI:** 10.1111/plb.70096

**Published:** 2025-09-01

**Authors:** C. Catalano, M. Di Guardo, M. Cortese, M. Caruso, S. La Malfa, G. Distefano, A. Gentile

**Affiliations:** ^1^ Department of Agriculture Food and Environment University of Catania Catania Italy; ^2^ Council for Agricultural Research and Economics Research Centre for Olive, Fruit and Citrus Crops Acireale Italy

**Keywords:** citrus, histology, segregating populations, tracheomycosis, vessel

## Abstract

- In plants, xylem is directly involved in conveyance of water and dissolved minerals, mechanical support of the plant, and tolerance to drought stress. Moreover, for several fruit crops affected by vascular diseases, an association between the morphology of xylem vessels and susceptibility was described. In fact, compartmentalization represents a key determinant mechanism of plant resistance to vascular infections. Mal secco is a severe tracheomycosis affecting many citrus species of relevant economic importance. Currently, both chemical and agronomic measures are not sufficient to contain the diffusion of the pathogen raising the interest for the elucidation of the host tolerance mechanism against mal secco.
- This study investigated the constitutive morphology of xylem tissue, in terms of vessel diameter and vessel density in 28 citrus genotypes, all characterized by a different degree of tolerance/susceptibility towards the disease. One‐year‐old stems, in three replicates per each genotype, were cut into 50‐μm sections and observed under an optical microscope after staining in safranine‐O.
- Analysis revealed a positive correlation between xylem vessel density and susceptibility to mal secco disease.
- These findings suggest that the constitutive morphology of xylem tissue could play a role in tolerance to mal secco, even though other mechanisms, both at histological and biochemical level, need to be unlocked and better elucidated.

## Graphical abstract

In citrus genotypes, xylem vessel density is highly correlated with susceptibility to mal secco disease caused by *Plenodomus tracheiphilus*.

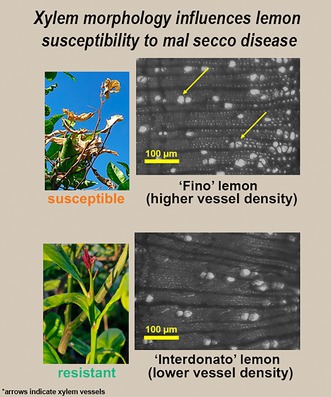

## INTRODUCTION

Among *Citrus* species of economic importance, lemon (*Citrus limon* (L.) Burm. *f*.) is the most susceptible to mal secco disease, a tracheomycosis caused by the mitosporic fungus *Plenodomus tracheiphilus* (Petri) Gruyter, Aveskamp and Verkley. The pathogen penetrates plants through wounds and colonizes xylem vessels, compromising water and solute transport (Nigro *et al*. 2011). The main symptoms are vein leaf chlorosis, wood discoloration, and the progressive desiccation of branches and shoots. Growers mainly rely on host tolerance to the disease, since both agronomic and biological strategies of control are not fully effective in limiting the spread of infections (Catalano *et al*. 2021).

Since establishment of the disease in the Mediterranean lemon‐producing countries, structural mechanisms of resistance have been investigated. As far as histology is concerned, alterations that more occur frequently after *P. tracheiphilus* infection are hyperplasia and hypertrophy of the xylem parenchyma cells, plasmolysis and necrosis of the cambium, gumming and crushing of the vessels, cell wall modifications, derangement of plastids, and increased number of mitochondria (Perrotta *et al*. 1979; Bassi *et al*. 1980).

As far as we know, the study of xylem vessel features in relation to susceptibility towards mal secco disease is limited and dated. In 1980, Lanza *et al*. (1980) exploited xylem anatomy and water conductivity in lemon varieties with different behaviour towards mal secco disease, but no correlation was found for xylem anatomy and susceptibility to mal secco disease, leading the authors to exclude analysis of these traits for the screening of lemon varieties with enhanced tolerance to the disease.

In contrast, several studies have demonstrated the influence of xylem vessel characteristics on the susceptibility of grapevine, elm, and avocado towards Petri and esca disease, Dutch elm disease and laurel disease (Solla & Gil 2002; Pouzoulet *et al*. 2020; Beier *et al*. 2021; Ramsing *et al*. 2021, respectively). In particular, xylem vessel morphology influences the efficiency of compartmentalization processes implemented by plants as defence responses against pathogen invasion, with a positive effect on disease severity and on plant ability to react to the infection by producing new healthy shoots (Pouzoulet *et al*. 2020).

Considering previous observations in citrus at the histological level during mal secco infections, and according to the promising recent results outlined above in other tree crops, the goal of this study was to investigate, in depth, the constitutive (pre‐infection) xylem vessel morphology and its influence on susceptibility towards mal secco disease.

## MATERIAL AND METHODS

A synthetic scheme of the Methods followed in the present research is provided in Figure S1.

### Plant material

The list of the 28 accessions analysed in the present study is provided in Table S1. This includes eight citrus genotypes whose susceptibility to mal secco is widely known and well documented according to field observation: the susceptible ‘Femminello’ lemon, ‘Fino’ lemon, ‘Cedro di Calabria’ citron (*C. medica*), and the field‐tolerant ‘Interdonato’ lemon, Seville sour orange (*C. aurantium*), ‘Shamouti’ sweet orange (*C. sinensis*), ‘Meyer’ lemon (*C. limon* var. *meyerii*), and the Khasi papeda (*C. latipes*) (Nigro *et al*. 2011; Russo *et al*. 2020). Overall, citrus accessions are considered susceptible to mal secco disease when they exhibit severe symptoms, whereas the accessions classified as resistant in the present work typically display no symptoms or only very mild symptoms under field conditions (Russo *et al*. 2020). In this study, hybrids were also considered from two segregating populations obtained in 2018 at the University of Catania and the Council for Agricultural Research and Economics, Research Center for Olive, Fruit, and Citrus Crops (CREA‐OFA), generated with the main aim of identifying molecular markers and candidate genes strongly associated with resistance towards mal secco disease (Catalano *et al*. 2021). Segregating individuals were obtained by crossing the resistant female parents ‘Interdonato’ lemon (POP1) and Khasi papeda (*C. latipes*, POP2) with the susceptible male parent ‘Femminello Siracusano *2Kr*’ lemon, characterized by high yield and outstanding fruit quality. These accessions were already characterized for their behaviour towards mal secco disease in previous studies (di Guardo *et al*. 2023; Arlotta *et al*. 2024). Briefly, phenotyping was performed both under natural infection of the pathogen, since experimental farms of both research institutions are located in areas characterized by the high pathogen pressure, and in controlled conditions with artificial inoculation. Ten accessions were selected from each population among those showing, respectively, the highest and the lowest level of susceptibility according to phenotyping performed both in field and *in planta* assays. The phenotyping dataset was defined by assigning score 2 for susceptible accessions and score 1 to resistant accessions. The mal secco‐susceptible accessions of POP1 were I129, I92, I135, I30, and I93, while resistant accessions were I21, I71, I82, I28, I128. For POP2, susceptible accessions were L47, L120, L114, L77, and L33, while L10, L20, L100, L176, and L98 were considered resistant. For the citrus accessions and cultivars, all samples were collected from 15‐year‐old plants from the citrus orchards of the Agricultural Research Organization (ARO) Volcani Center, Rishon LeTsiyon, Israel (31°98′72″N; 34°82′71″E). Stems of POP1 and POP2 were collected from 5‐year‐old plants grown in 50 L pots in a greenhouse at the experimental farm of the University of Catania (UNICT), located in the plain of Catania (37°24′33″N; 15°03′20″E), and in the screenhouse of the San Salvatore experimental farm of CREA‐OFA (37°37023″N; 15°09050″E), respectively. Considering the different growing conditions for the three groups of plants under analysis (citrus cultivars, POP1 and POP2), xylem vessel analysis was conducted separately.

### Xylem vessel analysis

Xylem vessel analyses were conducted on 1‐year‐old stems (8–10 mm Ø) as reported in Pouzoulet *et al*. (2020), with slight modifications. For each genotype, three stems were collected from three healthy plants (biological replicates) from different sides of the canopy. After sampling, stems were dehydrated in 70% ethanol overnight, then sections of 50 μm were cut with a rotary microtome (Reichert‐Jung Multicut 2045), stained with safranin‐O (0.2% alcohol solution) as contrast dye, and mounted on slides. Fifteen sections per stem were analysed, for a total of 45 sections per accession considered in the present analysis. In total, a minimum of 1500 vessels per genotype were considered for diameter measurement. Cross‐sections of the stems were visualized, and images were acquired under an optical microscope Leica DM2500 (Leica Microsystems, Wetzlar, Germany). The software ImageJ 1.53e was used to measure xylem vessel area and number (Schneider *et al*. 2012). Then, vessel density was calculated by dividing the number of vessels by the area observed (number of vessels mm^−2^), while vessel diameter (*d*) was obtained from the formula: *d* = √4A/π, assuming all xylem vessel sections as circles (equivalent circle diameter) (Scholz *et al*. 2013).

### Statistical analysis

Descriptive statistical analyses (mean, ± SD, ±SE) were performed using the ‘stat’ package of the R software (R Core Team 2021). ANOVA and Tukey's Honest Significant Difference test (*P*‐value <0.05) were performed for determining statistical differences between the studied accessions, while Pearson's correlation test was used to calculate the correlation coefficients and their significance by comparing the variables evaluated. All plots were generated through the ‘ggplot2’ packages of R software (Wickham 2016). Data were analysed considering separately the three groups of ‘citrus genotypes’, ‘POP1 individuals’, and ‘POP2 individuals’, since sampling for xylem analysis were performed on plant material cultivated under different conditions (environment, rootstock, agronomic practices), thus not allowing any comparison between groups.

## RESULTS AND DISCUSSION

Following recent information on other tracheomycosis diseases affecting tree crops, we performed an extensive analysis of xylem vessel diameter and density on a subset of 28 citrus genotypes having different behaviours in response to mal secco disease, to determine whether xylem vessel morphology could affect susceptibility to *P. tracheiphilus*, as a pre‐infection trait determining susceptibility and lack of effective response to the pathogen.

**Fig. 1:**
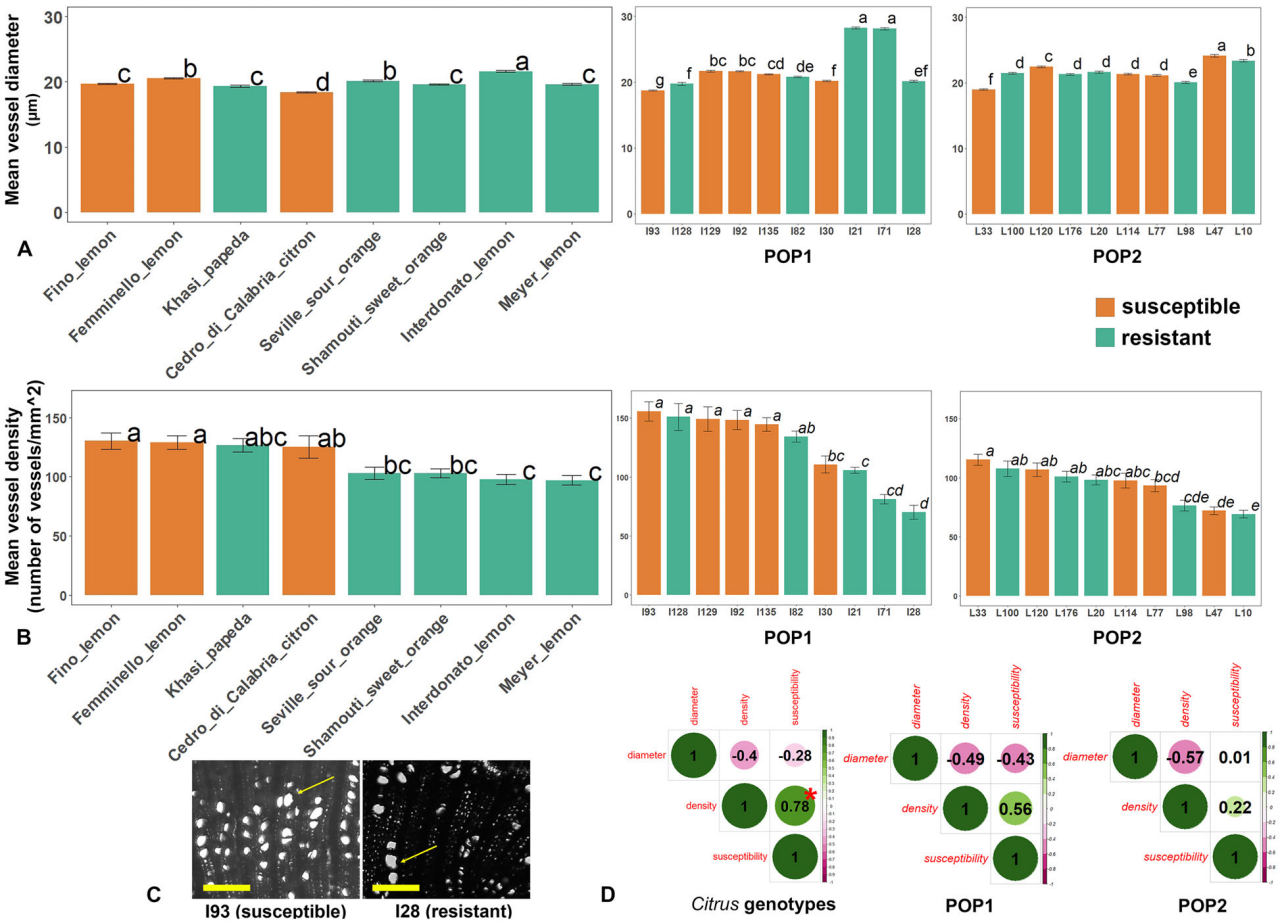
Mean vessel diameter (A) and mean vessel density (B) calculated for the genotypes under study. (C) cross‐sections of POP1 individuals showing opposing vessel density (Safranine‐O staining, scale bar = 100 μm, arrows indicate xylem vessels). (D) Correlation matrixes of the variables observed (orange bars indicate susceptible accessions and cultivars, green bars indicate resistant cultivars; different letters and asterisks indicate statistically significant differences at *P*‐value <0.001).

**Fig. 2:**
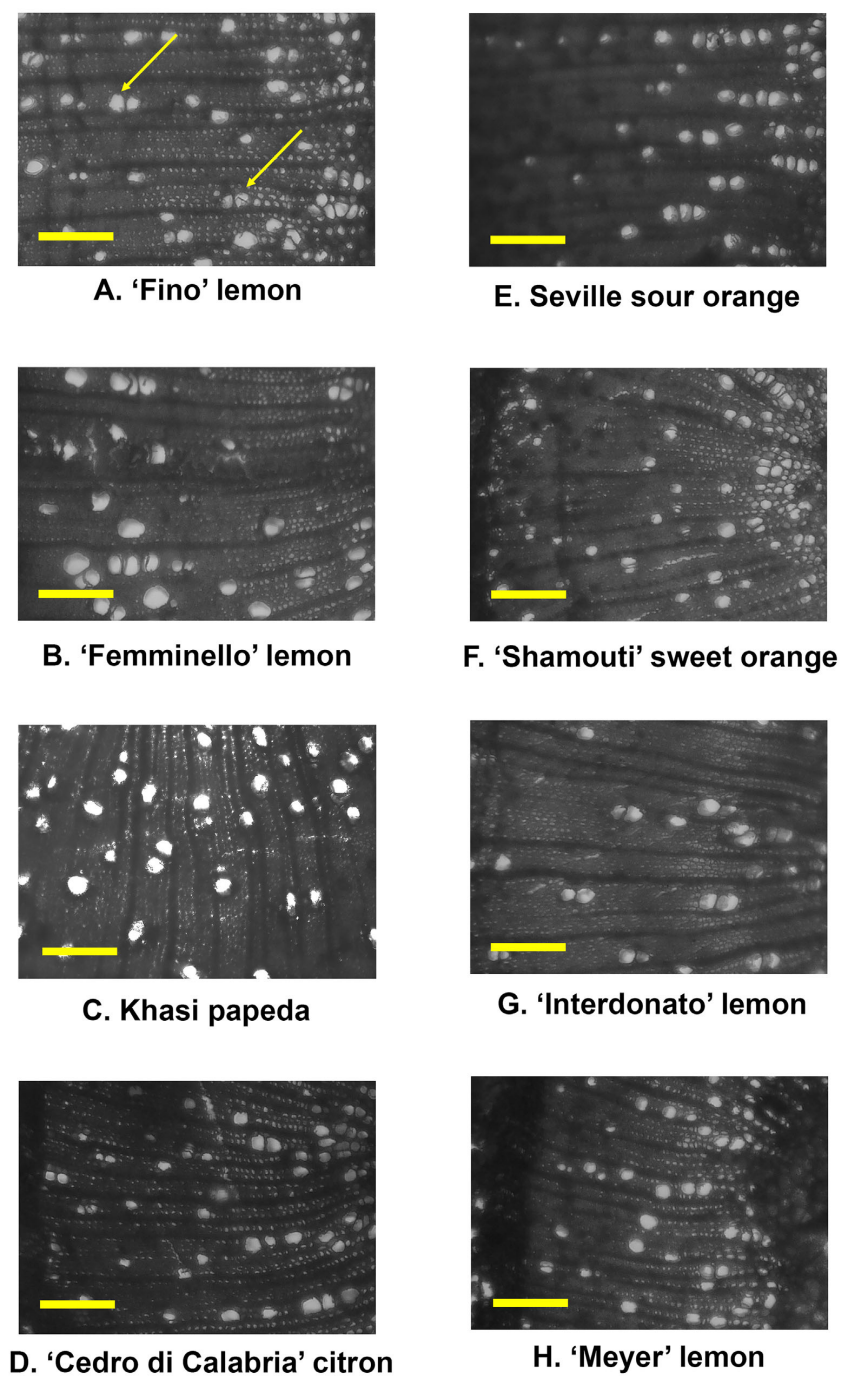
Cross‐sections of the citrus species analysed in this study, ordered according to results for vessel density analysis (A: ‘Fino’ lemon, B: ‘Femminello’ lemon, C: Khasi papeda, D: ‘Cedro di Calabria’ citron, E: Seville sour orange, F: ‘Shamouti’ sweet orange, G: ‘Interdonato’ lemon, H: ‘Meyer’ lemon) after safranine‐O staining. Scale bar = 100 μm, arrows indicate xylem vessels.

Both xylem vessel diameter and density were found to vary among the genotypes analysed (Figs 1 and 2). In terms of the vessel diameter, ‘Interdonato’ lemon had the highest average diameter (21.6 μm), followed by ‘Femminello’ lemon (20.5 μm), Seville sour orange (20.1 μm), ‘Fino’ lemon (19.7 μm), ‘Meyer’ lemon (19.6 μm), ‘Shamouti’ sweet orange (19.6 μm), Khasi papeda (19.3 μm), and citron (18.4 μm) (Fig. 1A). On the other hand, ‘Fino’ lemon had the highest xylem density, with 130.3 vessels mm^−2^, followed by ‘Femminello’ lemon (128.9 vessels mm^−2^), Khasi papeda (126.7 vessels mm^−2^), citron (125.4 vessels mm^−2^), Seville sour orange (103.1 vessels mm^−2^), ‘Shamouti’ sweet orange (102.9 vessels mm^−2^), ‘Interdonato’ lemon (97.9 vessels mm^−2^), and ‘Meyer’ lemon (97.1 vessels mm^−2^) (Figs 1B and 2). A high‐positive and statistically significant correlation was found between susceptibility and mean vessel density (0.78, *P*‐value <0.05) (Fig. 1D).

With special attention focused on ‘Femminello’ and ‘Interdonato’, being parental lines of POP1, the results indicate statistically significant differences (*P*‐value <0.001) for both vessel diameter, 20.6 μm and 21.6 μm, respectively, and vessel density, 128.9 and 97.9 vessels mm^−2^, respectively (Fig. 1A,B).

These genotypes were chosen as parentals of POP1, and the assumption of the segregation of this trait in the full‐sib population was confirmed. In fact, in individuals of POP1, vessel diameter ranged from 28.2 μm in the resistant genotype I21, to 18.7 μm in the susceptible genotype I93, which had the highest vessel density (155.6 vessels mm^−2^), while the lowest diameter was in the resistant I28 (70.3 vessels mm^−2^) (Fig. 1A–C). This difference might significantly affect the pathogen's ability to spread through the plant vascular system. In fact, this fungus can move actively in a radial direction through pit membranes, and mainly phialoconidia propagules then spread along the xylem tissue (Perrotta *et al*. 1979; Kroitor‐Keren *et al*. 2013). Therefore, having a higher number of vessels enables pathogen radial spread through the vascular system. Overall, these findings highlight the potential impact of xylem architecture on disease susceptibility. In POP1, there was a positive correlation between susceptibility and vessel density (0.56), and a negative correlation with vessel diameter (−0.43), although without any statistical significance in either case (Fig. 1D).

Considering the time‐ and cost‐effectiveness of the method used, this analysis is affordable and could be extended to a much larger number of lemon accessions and hybrids as a selection criterion, as also proposed for elm (Beier & Blanchette 2020). Moreover, analysis of xylem features in the whole segregating population might prove to be a useful strategy to investigate more in‐depth plant–pathogen interactions at multiple levels, and could be implemented in the QTL analysis performed on the selected germplasm to reveal the genetic determinants of resistance towards mal secco disease.

In terms of individuals of POP2, vessel diameter ranged from 24.1 μm in the susceptible genotype L47, to 18.9 μm in the susceptible genotype L33, which had the highest vessel density (115.3 vessels mm^−2^), while the lowest density was in the resistant L10 (69.2 vessels mm^−2^) (Fig. 1A,B). In POP2, there was a weak but positive non‐significant correlation between susceptibility and vessel density (0.21), but no correlation with vessel diameter (Fig. 1D). In this case, the lack of correlation may be attributed to behaviour of the parental lines of this population, Khasi papeda (*C. latipes*) and lemon ‘Femminello’, which exhibited statistically significant differences in xylem vessel diameter but not in xylem vessel density. POP2 was generated using *C. latipes* as a resistant parent, which belongs to a different subgenus (*Papeda*), and is phylogenetically distant from *C. limon* (Swingle 1943). The low correlation coefficient for POP2 leaves the discussion open into different strategies of tolerance to mal secco infection (e.g., biochemical modifications) that could be implemented differently among the huge number of accessions in the genus *Citrus*. In this case, other unknown mechanisms, rather than xylem morphology, likely play a major role in response to this pathogen.

Overall, the most relevant results from our analysis revealed a positive correlation in the citrus accessions between vessel density and susceptibility towards mal secco disease. In fact, the citrus accessions with the highest vessel density are considered most susceptible to mal secco disease. Since the fungus is able to actively move in a radial direction through the xylem pits or by attacking the primary cell wall (Perrotta *et al*. 1979), and mainly phialoconidia propagules spread through the xylem tissue (Kroitor‐Keren *et al*. 2013), we speculate that a lower vessel density could represent an effective morphological feature in limiting pathogen movement into the host tree. This hypothesis is mainly supported by the eight citrus accessions analysed rather than the 20 genotypes obtained through crossbreeding (POP1 and POP2). Further experiments and analysis on vulnerability to cavitation and the degree of vessel grouping will improve understand of differences between the studied accessions in behaviour towards mal secco disease. For instance, more tightly grouped vessels or lower vulnerability to cavitation should limit pathogen spread by restricting continuous xylem pathways, thereby enhancing plant tolerance to tracheomycosis. Therefore, taking both the results reported in the present study and those reported previously, xylem vessel constitutive morphology proved to be an interesting trait for further in‐depth investigation that could unlock the *P. tracheiphilus*–citrus interaction process and provide effective strategies to enhance host tolerance towards mal secco disease.

## CONCLUSION

Broadening the scope of investigation into the multiple factors determining resistance to a complex disease, such as tracheomycosis, we investigated xylem vessel diameter and density in 28 genotypes with opposing behaviours in response to mal secco disease. A high positive correlation was found between vessel density and susceptibility towards mal secco disease, especially in the studied eight citrus accessions. This represents new insights with respect to future studies of the lemon–*P. tracheiphilus* interaction, and a new possible trait of interest for use in selecting lemon breeding for resistance towards mal secco disease. Nonetheless, further research is still needed to better elucidate mechanisms responsible for this trait of interest, and to assess the physiological‐ and susceptibility‐related implications arising from differences in xylem morphology.

## CONFLICT OF INTEREST STATEMENT

The authors declare that the research was conducted in the absence of any commercial or financial relationships that could be construed as a potential conflict of interest.

## AUTHOR CONTRIBUTIONS

CC: conceptualization, investigation, formal analysis and writing – original draft. MDG, SLM, GD: writing – review and editing. MC: investigation, writing – review and editing. MC: resources, writing – review and editing. AG: resources, supervision, writing – review and editing.

## FUNDING INFORMATION

This research was founded by: Agritech National Research Center (European Union Next‐Generation EU, PIANO NAZIONALE DI RIPRESA E RESILIENZA, PNRR – MISSIONE 4 COMPONENTE 2, INVESTIMENTO 1.4—D.D. 1032 17/06/2022, CN00000022, CUP: E63C22000960006) Spoke 2 (Task 2.2.1: ‘Improved genetic materials to reduce the use of agrochemicals’), and AGRIVITA project “Difesa degli Agrumeti Italiani dal Malsecco – AGRIVITA”, CUP: C83C23000650006, Ministero dell'agricoltura, della sovranità alimentare e delle foreste (MASAF).

## Supporting information


**Supplementary Table 1.** List of the genotypes analyzed in the study.
**Supplementary Figure 1.** Workflow of the methods adopted in the study.
