# Supplementary material for: Xylem morphology influences lemon susceptibility to mal secco disease
Source: Plant Biol (Stuttg). 2025 Sep 1;27(7):1414–9. doi: 10.1111/plb.70096 (PMC12631519; doi:10.1111/plb.70096)
Supplement: Supplementary file 1 — Supplementary Table 1. List of the genotypes analyzed in the study. Supplementary Figure 1. Workflow of the methods adopted in the study. [file PLB-27-1414-s001.docx]

# Supporting information

**Supplementary Table 1.** List of the genotypes analyzed in the study.

| **Group** | **Accession** | **Species** | **Behavior towards mal secco disease** | **Reference** |
| --- | --- | --- | --- | --- |
| citrus variety | ‘Femminello’ lemon | *C. limon* Burm *f.* | susceptible | (Russo et al. 2020) |
| citrus variety | ‘Fino’ lemon | *C. limon* Burm *f.* | susceptible | (Russo et al., 2020) |
| citrus variety | ‘Interdonato’ lemon | *C. limon* Burm *f.* | resistant | (Russo et al. 2020) |
| citrus rootstock | Seville sour orange | *C. aurantium* | resistant | (Russo et al. 2020) |
| citrus variety | ‘Cedro di Calabria’ citron | *C. medica* | susceptible | (Russo et al. 2020) |
| citrus variety | ‘Shamouti’ sweet orange | *C. sinensis* | resistant | (Russo *et al.*, 2020) |
| citrus variety | ‘Meyer’ lemon | *C. limon* var. *meyerii* | resistant | (Russo et al. 2020) |
| citrus relative | Khasi papeda | *C. latipes* | resistant | (Russo et al. 2020) |
| POP1 | I129 | *C. limon* ‘Interdonato’ x *C. limon* ‘Femminello Siracusano *2Kr*’ | susceptible | (Di Guardo *et al.*, 2023) |
| POP1 | I92 | *C. limon* ‘Interdonato’ x *C. limon* ‘Femminello Siracusano *2Kr*’ | susceptible | (Di Guardo *et al.*, 2023) |
| POP1 | I135 | *C. limon* ‘Interdonato’ x *C. limon* ‘Femminello Siracusano *2Kr*’ | susceptible | (Di Guardo *et al.*, 2023) |
| POP1 | I30 | *C. limon* ‘*Interdonato’ x C. limon* ‘Femminello Siracusano *2Kr*’ | susceptible | (Di Guardo *et al.*, 2023) |
| POP1 | I93 | *C. limon* ‘Interdonato’ x *C. limon* ‘Femminello Siracusano *2Kr*’ | susceptible | (Di Guardo *et al.*, 2023) |
| POP1 | I21 | *C. limon* ‘Interdonato’ x *C. limon* ‘Femminello Siracusano *2Kr*’ | resistant | (Di Guardo *et al.*, 2023) |
| POP1 | I71 | *C. limon* ‘Interdonato’ x *C. limon* ‘Femminello Siracusano *2Kr*’ | resistant | (Di Guardo *et al.*, 2023) |
| POP1 | I82 | *C. limon* ‘Interdonato’ x *C. limon* ‘Femminello Siracusano *2Kr*’ | resistant | (Di Guardo *et al.*, 2023) |
| POP1 | I28 | *C. limon* ‘Interdonato’ x *C. limon* ‘Femminello Siracusano *2Kr*’ | resistant | (Di Guardo *et al.*, 2023) |
| POP1 | I128 | *C. limon* ‘Interdonato’ x *C. limon* ‘Femminello Siracusano *2Kr*’ | resistant | (Di Guardo *et al.*, 2023) |
| POP2 | L47 | Khasi papeda *C. latipes* x *C. limon* ‘Femminello Siracusano *2Kr*’ | susceptible | (Arlotta *et al.*, 2024) |
| POP2 | L120 | Khasi papeda *C. latipes* x *C. limon* ‘Femminello Siracusano *2Kr*’ | susceptible | (Arlotta *et al.*, 2024) |
| POP2 | L114 | Khasi papeda *C. latipes* x *C. limon* ‘Femminello Siracusano *2Kr*’ | susceptible | (Arlotta *et al.*, 2024) |
| POP2 | L77 | Khasi papeda *C. latipes* x *C. limon* ‘Femminello Siracusano *2Kr*’ | susceptible | (Arlotta *et al.*, 2024) |
| POP2 | L33 | Khasi papeda *C. latipes* x *C. limon* ‘Femminello Siracusano *2Kr*’ | susceptible | (Arlotta *et al.*, 2024) |
| POP2 | L10 | Khasi papeda *C. latipes* x *C. limon* ‘Femminello Siracusano *2Kr*’ | resistant | (Arlotta *et al.*, 2024) |
| POP2 | L20 | Khasi papeda *C. latipes* x *C. limon* ‘Femminello Siracusano *2Kr*’ | resistant | (Arlotta *et al.*, 2024) |
| POP2 | L100 | Khasi papeda *C. latipes* x *C. limon* ‘Femminello Siracusano *2Kr*’ | resistant | (Arlotta *et al.*, 2024) |
| POP2 | L176 | Khasi papeda *C. latipes* x *C. limon* ‘Femminello Siracusano *2Kr*’ | resistant | (Arlotta *et al.*, 2024) |
| POP2 | L98 | Khasi papeda *C. latipes* x *C. limon* ‘Femminello Siracusano *2Kr*’ | resistant | (Arlotta *et al.*, 2024) |


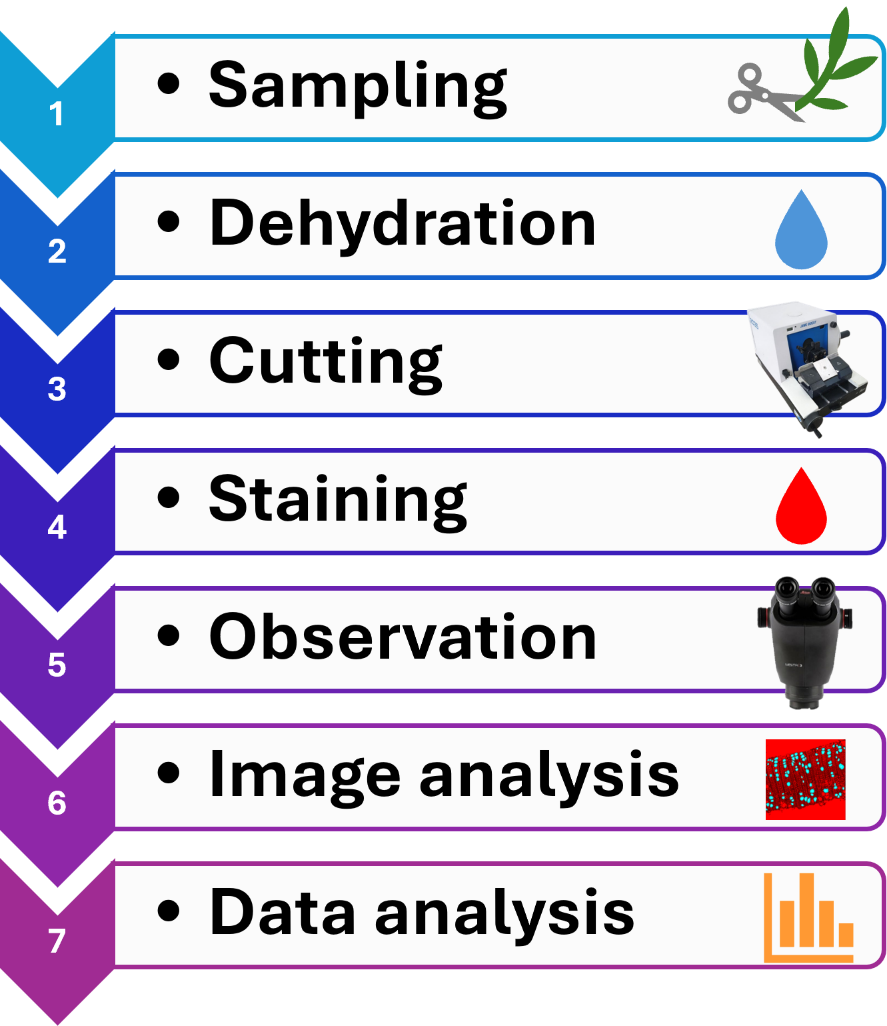


**Supplementary Figure 1.** Workflow of the methods adopted in the study. Xylem vessel analyses were conducted on one-year-old stems (8-10 mm Ø) as reported in Pouzoulet et al. (2020) with slight modifications. Per each genotype, three stems were collected from three healthy plants (biological replicates) from different sides of the canopy (1). After sampling, stems were dehydrated in 70% ethanol overnight (2) and then sections of 50 µm were cut with a rotary microtome (Reichert-Jung Multicut 2045) (3), stained with safranin-O (0.2% alcohol solution) as contrast dye and mounted on slides (4). Fifteen sections per stem were analysed, for a total of 45 sections per accession considered in the present analysis (5). On the whole, a minimum of 1500 vessels per genotype were considered for diameter measurement. Cross-sections of the stems were visualized, and images were acquired under the optical microscope Leica DM2500 (Leica Microsystems, Wetzlar, Germany). The software ImageJ 1.53e was used for measuring xylem vessel area and number (Schneider, Rasband and Eliceiri, 2012) (6). Then, vessel density was calculated by dividing the number of vessels by the area observed (number of vessels/mm2), while vessel diameter (d) was obtained from the formula: d = √4A/ π, assuming all xylem vessel sections as circles (equivalent circle diameter) (Scholz *et al.*, 2013). Descriptive statistical analyses (mean, standard deviation, standard error) were performed using the ‘stat’ package of the R software (R Core Team, 2021) (6). ANOVA and Tukey's Honest Significant Difference test (p value < 0.05) were performed for determining statistical difference between the genotypes under study, while Pearson’s correlation test was performed for calculating correlation coefficient and their significance by comparing the variables under evaluation. All plots were generated through the ‘ggplot2’ packages of the R software (Wickham, 2016). Data were analysed considering separately the three groups of ‘citrus genotypes’, ‘POP1 individuals’ and ‘POP2 individuals’, since sampling for xylem analysis were performed on plant material cultivated in different conditions (environment, rootstock, agronomical practices), thus not allowing any comparison between groups.

## References for Material and Methods

Arlotta, C. *et al.* (2024) ‘Phenotypic Evaluation of a Lemon Hybrid Population to Identify Sources of Resistance to Plenodomus tracheiphilus’, *HortScience*, 59(5), pp. 658–665. doi: 10.21273/HORTSCI17673-23.

Di Guardo, M. *et al.* (2023) ‘De novo assembly of Citrus limon and target-sequence genotyping toward the detection of genes involved in tolerance to “mal secco” disease’, in *Acta Horticulturae*. doi: 10.17660/ActaHortic.2023.1362.31.

Pouzoulet, J. *et al.* (2020) ‘Behind the curtain of the compartmentalization process: Exploring how xylem vessel diameter impacts vascular pathogen resistance’, *Plant, Cell & Environment*, 43(11), pp. 2782–2796. doi: 10.1111/pce.13848.

R Core Team (2021) ‘R core team (2021)’, *R: A language and environment for statistical computing. R Foundation for Statistical Computing, Vienna, Austria. URL http://www. R-project. org*.

Russo, R. *et al.* (2020) ‘Identification of Field Tolerance and Resistance to Mal Secco Disease in a Citrus Germplasm Collection in Sicily’, *Agronomy*, 10, p. 1806. doi: 10.3390/agronomy10111806.

Schneider, C. A., Rasband, W. S. and Eliceiri, K. W. (2012) ‘NIH Image to ImageJ: 25 years of image analysis’, *Nature Methods*. doi: 10.1038/nmeth.2089.

Scholz, A. *et al.* (2013) ‘How to quantify conduits in wood?’, *Frontiers in Plant Science*, 4. doi: 10.3389/fpls.2013.00056.

Wickham, H. (2016) *ggplot2: Elegant Graphics for Data Analysis. Springer-Verlag New York*, *Media*.
